# Tetra­methyl­ammonium borohydride from powder data

**DOI:** 10.1107/S1600536811029291

**Published:** 2011-07-30

**Authors:** Tomasz Jaroń, Wojciech Grochala

**Affiliations:** a Faculty of Chemistry, University of Warsaw, Pasteura 1, 02093 Warsaw, Poland; b ICM, University of Warsaw, Pawińskiego 5a, 02106 Warsaw, Poland

## Abstract

In the crystal structure of the title compound, C_4_H_12_N^+^·BH_4_^−^, the tetra­methyl­ammonium cations are situated on special positions with site symmetry 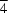 *m*2. The borohydride anions are situated on special positions with 4*mm* site symmetry and show rotational disorder around the fourfold axis.

## Related literature

For details of the synthesis, see: Banus *et al.* (1952 ▶); King *et al.* (1956 ▶). For previous studies of the title compound, see: Harmon *et al.* (1974 ▶); Eckert *et al.* (2004 ▶). The isostructural compounds (CH_3_)_4_NClO_4_ and (CH_3_)_4_NBF_4_ were reported by McCullough (1964 ▶) and Giuseppetti *et al.* (1992 ▶), respectively. For applications of the title compound, see: Evans *et al.* (1988 ▶).
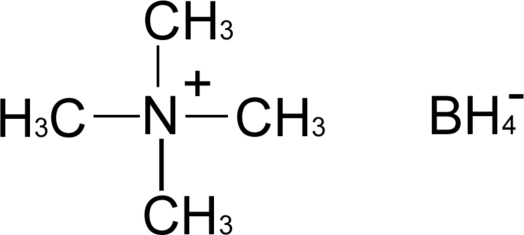


## Experimental

### 

#### Crystal data

C_4_H_12_N^+^·BH_4_^−^
*M* *_r_* = 88.99
Tetragonal, 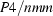

*a* = 7.9133 (2) Å
*c* = 5.65696 (17) Å
*V* = 354.24 (2) Å^3^
*Z* = 2
Cu *K*α radiation, λ = 1.54051, 1.54433 Å
μ = 0.33 mm^−1^
*T* = 298 K
cylinder, 18 × 1 mm

#### Data collection

Bruker D8 Discover diffractometer
Specimen mounting: quartz capillary
Data collection mode: transmission
Scan method: continuous
2θ_min_ = 8°, 2θ_max_ = 121°, 2θ_step_ = 0.012°

#### Refinement

*R*_p_ = 0.014
*R*_wp_ = 0.020
*R*_exp_ = 0.007
*R*_Bragg_ = 0.053
χ^2^ = 7.673
9220 data points
56 parameters
14 restraints
H atoms treated by a mixture of independent and constrained refinement

### 

Data collection: *DIFFRACplus* (Bruker, 2006 ▶); cell refinement: *X-CELL* (Neumann, 2003 ▶) and *JANA2006* (Petricek *et al.*, 2006 ▶); data reduction: *DIFFRACplus*; program(s) used to solve structure: *JANA2006*; program(s) used to refine structure: *JANA2006*; molecular graphics: *CrystalMaker* (Palmer, 2005 ▶); software used to prepare material for publication: *publCIF* (Westrip, 2010 ▶).

## Supplementary Material

Crystal structure: contains datablock(s) global, I. DOI: 10.1107/S1600536811029291/cv5120sup1.cif

Supplementary material file. DOI: 10.1107/S1600536811029291/cv5120Isup2.cml

Rietveld powder data: contains datablock(s) I. DOI: 10.1107/S1600536811029291/cv5120Isup2.rtv

Additional supplementary materials:  crystallographic information; 3D view; checkCIF report

**Fig. 1.**
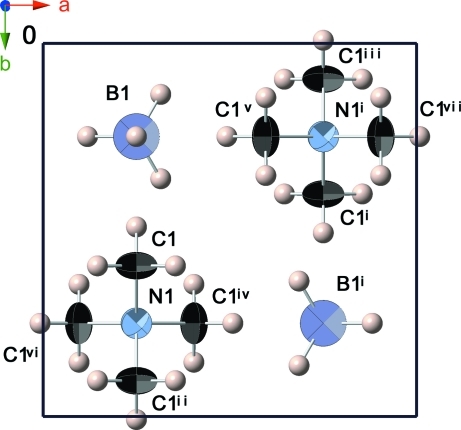
A content of the unit cell of (I) viewed along axis c and showing the atomic labelling and 50% probabilty displacement ellipsoids [symmetry codes: (i) -x + 1,-y + 1,-z + 1; (ii) -x + 1/2,-y + 3/2,z; (iii) x + 1/2,y - 1/2,-z + 1; (iv) -y + 1,x + 1/2,-z + 1; (v) y,-x + 1/2,z; (vi) y - 1/2,-x + 1,-z + 1; (vii) -y + 3/2,x,z]. The H atoms are shown as spheres of arbitrary radius. Only one orientation of disordered BH4 anions is shown for clarity.

Enhanced figure: interactive version of Fig. 1

## Figures and Tables

**Fig. 2.**
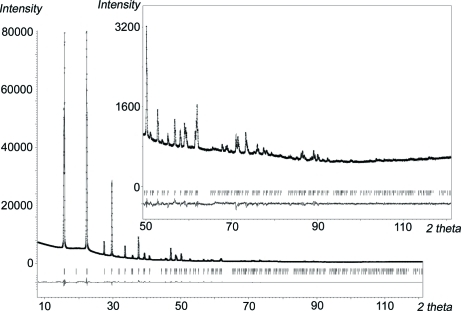
The results of Rietveld refinement. Measured data are given as asterisks, the calculated profile as a solid line, and the difference profile as a solid line below. Vertical markers above the difference profile indicate the calculated Bragg reflection positions. The insert shows high-angle part magnified ca 18 times.

## supplementary crystallographic information

### Comment

Tetramethylammonium borohydride (I) and its derivatives have been used as selective reductors in organic chemistry (Evans *et al.*, 1988) or as a source of hydrogen-rich BH_4_^-^ anions for inorganic synthesis.

The structure of compound (I) has not been reported before; only the unit-cell parameters (a = 7.29 Å, c = 5.696 Å), the tentative space group (P4/n) (King *et al.*, 1956) and several interatomic distances (Eckert *et al.*, 2004) have been given. The structure presented here is isomorphous to the ambient temperature structures of (CH_3_)_4_NBF_4_ (Giuseppetti *et al.*, 1992) and (CH_3_)_4_NClO_4_ (McCullough, 1964) and is composed of distinct (CH_3_)_4_N^+^ cations and BH_4_^-^ anions.

The central atoms of the ions are separated by d(N1, B1) = 4.537 (4) Å, which compares well with the shortest N—B distances seen for fluoroborate (4.79 Å, s.u. not given in the paper) and N—Cl distances in perchlorate (4.86 Å, s.u. not given in the paper). Methyl groups in (I) are ordered (in contrast to (CH_3_)_4_NClO_4_, where the hydrogen positions are disordered) and arranged in a staggered conformation like in (CH_3_)_4_NBF_4_. This is explained by an increasing separation of cations as measured by d(N—N') distance of 5.5955 (2), 5.82 (s.u. not given in the paper), and 5.90 (s.u. not given in the paper) for (I), (CH_3_)_4_NBF_4_, and (CH_3_)_4_NClO_4_, respectively. The borohydride anions are centred at 2c (4 mm) site with B1 and H3 atoms at the fourfold symmetry axis, while H4 and H5 are used for the representation of disorder (four BH_4_^-^ tetrahedra sharing vertex of H3 can be constructed). The B—H infrared absorption bands (δ_H—B—H_ = 1072 cm^-1^, ν_B—H_ = 2225 cm^-1^ and 2288 cm^-1^) are very broad, up to *ca* 800 cm^-1^ for stretching bands (!), which confirms H disorder of the BH_4_^-^ anions in compound (I) (Harmon *et al., 1974*). In such arrangement the closest distances between hydrogen atoms of (CH_3_)_4_N^+^ and BH_4_^-^ are: d(H1—H3) = 2.39 (2) Å, d(H2—H4) = 2.47 (3) Å and d(H2—H5)= 2.49 (3) Å, thus above the maximum range of a typical dihydrogen bond length of 2.2 Å.

### Experimental

Compound (I) commercially available from Sigma-Aldrich (> 95%) has been used for powder XRD measurements without additional purification. The FTIR spectrum of compound (I) in KBr pellet has been recorded using Bruker Vertex 80v vacuum spectrometer.

### Refinement

The powder diffraction pattern of (I) was indexed with X-Cell (Neumann, 2003) in a tetragonal system of extinction class P4/nmm. The initial model of the structure of compound (I) was constructed according to the symmetry considerations, with all heavy atoms at special positions: N1 at 2 b (4*m*2), C1 at 8i (*m*), and B1 at 2c (4 mm). H1 and H2 (hydrogen atoms of the methyl groups) were placed at sites 8i (*m*), and 16k (1), respectively. An alternative structure with disordered methyl groups led to a worse Rietveld fit. Location of the hydrogen atoms of BH_4_^-^ group was more problematic, as the tetragonal axis is incompatible with the symmetry alements of tetrahedron. The model containing four overlaping BH_4_^-^ tetrahedra has been used for the Rietveld refinement performed using Jana2006 (Petricek *et al.* 2006). The following restraints have been aplied for the refinement: d(C1—H1), d(C1—H2) = 1.00 s.u. = 0.005 [Å], d(B1—H3) = 1.12, s.u. = 0.005 [Å], d(B1—H4), d(B1—H5) = 1.12, s.u. = 0.01 [Å]; a(Hi—C1—Hj), a(Hi—C1—N1) = 109.47, s.u.=0.01 [°], and a(H3—B1—H4), a(H3—B1—H5), a(H4—B1—H5) = 109.47, s.u.=0.01 [°]. The atomic displacement parameters (ADP) of hydrogen atoms were restricted according to the riding model to *U*_iso_H1 = *U*_iso_H2 = 1.2*U*_iso_C1 and *U*_iso_H3 = *U*_iso_H4 = *U*_iso_H5 = 1.5*U*_iso_B1.

### Crystal data

**Table:**

C_4_H_12_N^+^·BH_4_^−^	*F*(000) = 104
*M**_r_* = 88.99	*D*_x_ = 0.834 Mg m^−^^3^*D*_m_ = 0.813 Mg m^−^^3^*D*_m_ measured by helium pycnometry (Banus et al., 1952)
Tetragonal, *P*4/*n**m**m*	Cu *K*α radiation, λ = 1.54051, 1.54433 Å
Hall symbol: -P 4a 2a	µ = 0.33 mm^−^^1^
*a* = 7.9133 (2) Å	*T* = 298 K
*c* = 5.65696 (17) Å	white
*V* = 354.24 (2) Å^3^	cylinder, 18 × 1 mm
*Z* = 2	Specimen preparation: Prepared at 298 K

### Data collection

**Table:**

Bruker D8 Discover diffractometer	Data collection mode: transmission
none	Scan method: continuous
Specimen mounting: quartz capillary	2θ_min_ = 8°, 2θ_max_ = 120.999°, 2θ_step_ = 0.012°

### Refinement

**Table:**

*R*_p_ = 0.014	14 restraints
*R*_wp_ = 0.020	2 constraints
*R*_exp_ = 0.007	All H-atom parameters refined
*R*_Bragg_ = 0.053	Weighting scheme based on measured s.u.'s *w* = 1/[σ^2^(*I*) + 0.0016*I*^2^]
χ^2^ = 7.673	(Δ/σ)_max_ = 0.009
9220 data points	Background function: 25 Legendre polynoms
Profile function: Pseudo-Voigt	Preferred orientation correction: none
56 parameters	

### Fractional atomic coordinates and isotropic or equivalent isotropic displacement parameters (Å^2^)

**Table:**

	*x*	*y*	*z*	*U*_iso_*/*U*_eq_	Occ. (<1)
H4	0.315 (2)	0.363 (4)	0.957 (3)	0.14971 (4)*	0.25
H2	0.1457 (17)	0.5963 (4)	0.2429 (17)	0.10060 (2)*	
B1	0.25	0.25	0.8925 (14)	0.084 (5)	
H3	0.25	0.25	0.702 (4)	0.14971 (4)*	
C1	0.25	0.5959 (3)	0.3460 (5)	0.065 (2)	
H1	0.25	0.492 (2)	0.4477 (19)	0.10060 (2)*	
H5	0.25	0.119 (5)	0.957 (3)	0.14971 (4)*	0.25
N1	0.25	0.75	0.5	0.045 (3)	

### Atomic displacement parameters (Å^2^)

**Table:**

	*U*^11^	*U*^22^	*U*^33^	*U*^12^	*U*^13^	*U*^23^
B1	0.100 (7)	0.100 (7)	0.053 (9)	0	0	0
C1	0.084 (4)	0.036 (3)	0.075 (4)	0	0	−0.013 (3)
N1	0.045 (4)	0.045 (4)	0.045 (7)	0	0	0

### Geometric parameters (Å, °)

**Table:**

B1—H3	1.08 (2)	C1—H1	1.004 (15)
B1—H4	1.10 (3)	C1—H2	1.010 (12)
B1—H5	1.10 (4)	C1—N1	1.498 (3)
			
H1—C1—H2	109.5 (5)	H4—B1—H4^ii^	109.5 (16)
H2—C1—N1	109.5 (4)	H4—B1—H5^iii^	109.5 (12)
H2—C1—H2^i^	109.5 (9)	C1—N1—C1^iv^	108.91 (14)
H3—B1—H5	109.5 (12)	C1—N1—C1^v^	109.75 (7)
H4—B1—H3	109.5 (10)		

Symmetry codes: (i) −*x*+1/2, *y*, *z*; (ii) *x*, −*y*+1/2, *z*; (iii) *y*, −*x*+1/2, *z*; (iv) −*x*+1/2, −*y*+3/2, *z*; (v) *y*−1/2, *x*+1/2, −*z*+1.
